# Interaction between PPAR γ and SORL1 gene with Late-Onset Alzheimer’s disease in Chinese Han Population

**DOI:** 10.18632/oncotarget.15691

**Published:** 2017-02-25

**Authors:** Hui Zhang, Wei Zheng, Linlin Hua, Yutong Wang, Jinfeng Li, Hongying Bai, Shanshan Wang, Mingyao Du, Xuelian Ma, Chunyang Xu, Xiaodong Li, Bin Gong, Yunliang Wang

**Affiliations:** ^1^ Department of Neurology, The 148 Central Hospital of PLA, Shandong, China; ^2^ The Second Affiliated Hospital of Zhengzhou University, Zhengzhou, China; ^3^ Medical College of Henan University, Kaifeng, China; ^4^ Department of Neurology, The Second Affiliated Hospital of Zhengzhou University, Zhengzhou, China

**Keywords:** SORL1, PPAR G, single nucleotide polymorphism, alcohol drinking, interaction

## Abstract

**Aims:**

To investigate the impact of sortilin-related receptor 1 gene 1 (*SORL1)* and peroxisome proliferator activated receptor gamma (*PPAR G)* gene single nucleotide polymorphisms (SNPs), gene- gene and gene- environment interactions and haplotype on late-onset Alzheimer’s disease (LOAD) risk.

**Methods:**

Hardy-Weinberg equilibrium (HWE), haplotype analysis and pairwise linkage disequilibrium (LD) analysis were investigated by using SNPStats (available online at http://bioinfo.iconcologia.net/SNPstats). Logistic regression was performed to investigate association between SNPs and LOAD. Generalized multifactor dimensionality reduction (GMDR) was used to investigate the interaction among gene- gene and gene- environment interaction.

**Results:**

Logistic regression analysis showed that LOAD risk was significantly higher in carriers of the A allele of rs1784933 polymorphism than those with GG (GA+ AA versus GG), adjusted OR (95%CI) = 1.63(1.27-1.98), and higher in carriers of G allele of the rs1805192 polymorphism than those with CC (CG+ GG versus CC), adjusted OR (95%CI) = 1.70 (1.25-2.27). GMDR analysis suggested a significant two-locus model (*p* = 0.0010) involving rs1784933 and rs1805192, and a significant two-locus model (*p* = 0.0100) involving rs1784933 and alcohol drinking. Haplotype containing the rs1784933- A and rs689021- C alleles were associated with a statistically increased LOAD risk (OR = 1.86, 95%CI = 1.37– 2.52, *p* < 0.001).

**Conclusions:**

We conclude that rs1784933 and rs1805192 minor alleles, gene- gene interaction between rs1784933 and rs1805192, gene- environment interaction between rs1784933 and alcohol drinking, and haplotype containing the rs1784933- A and rs689021- C alleles are all associated with increased LOAD risk.

## INTRODUCTION

Alzheimer's disease (AD) was the main cause for dementia in persons with middle and old age [1], and is a complex and progressive neurodegeneration characterised by large numbers of senile plaques and neurofibrillary tangles in the brain [2]. Clinically, late-onset AD (LOAD) is more common type of AD and the heritability for susceptibility to LOAD could be 80% in previous studies [3]. The etiology and pathogenesis of LOAD are still not clear, AD was a multifactorial disease and the complex pathology was resulted by the interaction of both genetics and environmental factors, however, until recently, the only reliable risk factor, the e4 allele of apolipoprotein E (*APOE*) was verified. It was necessary to find and validate biomarkers for AD prevention, especially for LOAD, which has a strong genetic component [4], and several genes have been identified in the genome-wide association studies [5, 6], including the neuronal sortilin-related receptor (*SORL1*) [7] and peroxisome proliferator activated receptor gamma (*PPAR G*) gene [8].

*SORL1* gene locates on chromosome 11q23.2–q24.2 [9]. Recent studies and replication studies have indicated that polymorphisms within *SORL1* gene were associated with susceptibility to AD, which support the association between SNPs within *SORL1* gene and AD risk [10- 13]. However, the other studies concluded controversial results, which indicated a weak or no association between SNPs in *SORL1* gene and AD risk in Caucasian populations [14–16]. Recently, some studies reported that *PPAR G* can regulate amyloidogenic pathways [17, 18], they suggest that *PPAR G* may be a potential candidate gene for AD. However, results on association between *PPAR G* and LOAD were inconsistent yet [19, 20]. In addition, LOAD susceptibility could be influenced by both environmental and genetic factors, and their synergistic effects between gene and environment, and previous studies have suggested that alcohol drinking was an important risk factor of LOAD [21, 22]. However, till now, less study focused on gene- alcohol drinking interaction on LOAD risk.

In consideration of the previous inconsistent results on association of *PPAR G* and *SORL1* gene with LOAD, less numbers study on gene- alcohol drinking interaction and linkage disequilibrium (LD) among SNPs. In this study, we aimed to investigate the impact of *PPAR G* and *SORL1* gene SNPs, additional gene- gene, gene- environment interaction and haplotype combination on LOAD risk.

## MATERIALS AND METHODS

### Participants

In this case-control study, participants were consecutively recruited between January 2009 and November 2014 from the Second Affiliated Hospital of Zhengzhou University. Clinical diagnosis of probable AD is made according to the revised criteria of National Institute of Neurological and Communicative Disorders and Stroke/ Alzheimer's Disease and Related Disorders Association (NINCDS/ ADRDA) [23], participants with advanced, severe, progressive, or unstable infectious, metabolic, immunologic, endocrinological, hepatic, hematological, pulmonary, cardiovascular, gastrointestinal, and/or urological diseases are excluded. The detailed participant selection methods have been described in our previous study [24]. Data on demographic information, mini-mental state examination (MMSE), educational year, lifestyle risk factors, smoking and drinking status, prevalence of stroke, prevalence of diabetes and family history of AD for all participants are obtained using a questionnaire administered by trained staffs. Body weight, height and waist circumference (WC) are measured, and body mass index (BMI) are calculated. Blood samples are collected in the morning after at least 8 hours of fasting. All plasma and serum samples are frozen at –80°C until laboratory testing. Plasma glucose is measured using an oxidase enzymatic method. The concentrations of HDL cholesterol and triglycerides are assessed enzymatically using an automatic biochemistry analyzer (Hitachi Inc., Tokyo, Japan) and commercial reagents.

### Genomic DNA extraction and genotyping

SNPs within the *SORL1* and *PPAR G* gene were selected according to the following methods: 1) SNPs, which have been reported associations with AD and not been well studied; 2) SNPs, the MAF of which were more than 5%. At last, three SNPs of *SORL1* gene and three SNPs of *PPAR G* gene are selected for genotyping in the study, including: rs709158, rs1805192 and rs10865710 within *PPAR G* gene, rs1784933, rs3824966 and rs689021 within *SORL1* gene. Genomic DNA is extracted from EDTA-treated whole blood, using the DNA Blood Mini Kit (Qiagen, Hilden, Germany) according to the manufacturer's instructions. The genotyping methods for *PPAR G* gene have been described in our previous study [24]. Genotyping of three SNPs within *SORL1* gene were performed using polymerase chain reaction and restriction fragment length polymorphism (PCR-RFLP) analysis. PCR primer sequences for each polymorphism were shown in Table 1. The PCR reactions were carried out in a final volume of 25 μl containing: 10 × PCR buffer, 4.5 mM MgCl_2_ (Roche, Germany), 0.4 mM of each dNTP (Fermentas, Germany), 10 pmol of each primer, 30 ng template DNA, 1 U Taq DNA polymerase (Roche, Germany) and sterile distilled water up to 25 μl. PCR conditions were: 94 °C for 15min, then 45 cycles of 94 °C for 20 s, 56 °C for 30 s, and 72 °C for 1min, with a final extension at 72 °C for 30min and then reactions held at 4 °C.

**Table 1:** Description and Probe sequence for 6 SNPs used for Taqman fluorescence probe analysis

SNP ID	Chromosome	Functional Consequence	Major/ minor alleles	Nucleotide sequences/ Probe sequence
*SORL1*				
rs689021	11:121500411	Intron variant	T/C	Forward: ACGTTGGATGACCTTACAGATGATGCAGCC Reverse: ACGTTGGATGGGCCATAGTTTCCTAGCATC
rs3824966	11:121577474	Intron variant	G/C	Forward: ACGTTGGATGCCAAGCTAATTCTCAGAGCC Reverse: ACGTTGGATGTTGACAGCACTCATCCGTTC
rs1784933	11:121618707	Intron variant	G/A	Forward: ACGTTGGATGTTTGAAGCAGTTCCAGGGTC Reverse: ACGTTGGATGGAATGGAAGAGGACATCAGC
*PPARG*				
rs709158	3:12421677	Intron variant	A/G	5’-AGATACGGGGGAGGAAATTCACTGG[A/G] TTTTACAATATATTTTTCAAGGCAA-3’
rs10865710	3:12311699	Intron variant, upstream variant 2KB	C/G	5’-TTGGCATTAGATGCTGTTTTGTCTT[C/G] ATGGAAAATACAGCTATTCTAGGAT-3’
rs1805192	3:12379739	Missense	C/G	5’-ACCTCAGACAGATTGTCACGGAACA[C/T] GTGCAGCTACTGCAGGTGATCAAGA-3’

### Statistical analysis

The means and standard deviations (SD) were calculated for normally distributed continuous variables, and percentages were calculated for categorical variables. The categorical data were analyzed using χ^2^ test. Further, continuous variables were analyzed using Student's t test. Hardy-Weinberg equilibrium (HWE), haplotype analysis and pairwise LD analysis were investigated by using SNPStats (available online at http://bioinfo.iconcologia.net/SNPstats). Logistic regression was performed to investigate association between SNPs and LOAD. Generalized multifactor dimensionality reduction (GMDR) was used to investigate the interaction among gene- gene and gene- environment interaction, cross-validation consistency, the testing balanced accuracy, and the sign test, to assess each selected interaction were calculated. The cross-validation consistency score is a measure of the degree of consistency with which the selected interaction is identified as the best model among all possibilities considered. Testing-balanced accuracy is a measure of the degree to which the interaction accurately predicts case–control status, and yields a score between 0.50 (indicating that the model predicts no better than chance) and 1.00 (indicating perfect prediction). Finally, the sign test, or permutation test (providing empirical P-values), for prediction accuracy can be used to measure the significance of an identified model.

## RESULTS

A total of 880 participants (514 males, 366 females) were selected, including 430 LOAD patients and 450 control subjects. The mean age of all participants was 81.7 ± 15.9 years old. Table 2 shows the general characteristics, clinical and blood biochemical index for all participants. The cases have the higher alcohol- drinking rate than controls. The means of FPG and TG were significantly higher in cases and controls, but the mean of HDL was lower in cases and controls.

**Table 2:** General characteristics of 880 study participants in case and control group

Variables	Case group (*n*= 430)	Normal group (*n*= 450)	*p*-values
Age (year)	81.4±16.1	82.3±15.7	0.401
Males, N (%)	246 (57.2)	268(59.6)	0.480
Smoke, N (%)	151 (35.1)	145(32.2)	0.364
Alcohol consumption, N (%)	188 (43.7)	160 (35.6)	0.013
WC (cm)	89.2±19.8	87.7±19.4	0.257
BMI (kg/m^2^)	25.1±8.9	24.8±9.1	0.621
FPG (mmol/L)	5.8±1.6	5.5±1.9	0.012
TG (mmol/L)	1.4±0.8	1.3± 0.7	0.048
TC (mmol/L)	4.6±0.8	4.5±0.9	0.082
HDL (mmol/L)	1.21±0.65	1.34±0.63	0.002
Stroke	16 (3.72)	20 (4.44)	0.255
MMSE (scores)	15.16±5.51	29.12±4.97	<0.001
Diabetes	36 (8.37)	43(9.56)	0.539
Educational year	7.5±3.12	7.8±3.31	0.167

Note: Means± standard deviation for age, WC, BMI, FPG, TC, TG, HDL-C and ANP; TC, total cholesterol; HDL, high density lipoprotein; FPG, fast plasma glucose; TG, triglyceride; WC, waist circumference; BMI, body mass index; MMSE, mini-mental state examination;

In Table 3, the frequencies for the rs1784933- A allele within *SORL1* gene and rs1805192- G allele within *PPAR G* were significantly higher in LOAD cases than that in controls. The carriers with the rs1784933- A allele have higher LOAD risk than those with GG genotype (GA+ AA versus GG), adjusted OR (95%CI) = 1.63 (1.27-1.98), and the carriers with rs1805192- G allele also have higher LOAD risk than those with CC genotype (CG+ GG versus CC), adjusted OR (95%CI) = 1.70 (1.25-2.27). However, we the others SNP within *SORL1* and *PPAR G* gene were not associated with LOAD susceptibility after covariates adjustment.

**Table 3:** Genotype and allele frequencies of 6 SNPs between case and control group

Gene/ SNP	Genotypes and Alleles	Frequencies *N* (%)		OR(95%CI)*	*P*- values	HWE test for controls
		Control (*n*=450)	Case (*n*=430)			
*SORL1*						
rs689021	Co- dominant					
	TT	271 (60.2)	235(54.6)	1.00		0.360
	TC	152 (33.8)	153(35.6)	1.13 (0.82-1.85)	0.621	
	CC	27 (6.0)	42(9.8)	1.40 (0.89-1.98)	0.506	
	Dominant					
	TT	271 (60.2)	235(54.6)	1.00		
	TC +CC	179(39.8)	195 (45.4)	1.19 (0.83-1.89)	0.605	
	Allele, C (%)	206(22.9)	237(27.6)			
rs3824966	Co- dominant					
	GG	276(61.3)	243(56.5)	1.00		0.691
	GC	151(33.6)	156(36.3)	1.27(0.95-1.71)	0.236	
	CC	23(5.1)	31(7.2)	1.45(0.81-2.22)	0.379	
	Dominant					
	GG	276(61.3)	243(56.5)	1.00		
	GC+CC	174(38.7)	187(43.5)	1.42(0.92-1.87)	0.252	
	Allele, C (%)	197(21.9)	218(25.3)			
rs1784933	Co- dominant					0.999
	GG	288(64.0)	198(46.1)	1.00		
	GA	144(32.0)	182(42.3)	1.55(1.24-1.91)	<0.001	
	AA	18(4.0)	50(11.6)	2.08 (1.41-2.92)	<0.001	
	Dominant					
	GG	288(64.0)	198(46.1)	1.00		
	GA+AA	162(36.0)	232(53.9)	1.63(1.27-1.98)	<0.001	
	Allele, A (%)	180(20.0)	282(32.8)			
*PPAR G*						
rs1805192	Co- dominant					0.073
	CC	283(62.9)	212(49.3)	1.00		
	CG	139(30.9)	164(38.1)	1.57 (1.21-1.79)	<0.001	
	GG	28(6.2)	54(12.6)	2.15 (1.42-2.98)	<0.001	
	Dominant					
	CC	283(62.9)	212(49.3)	1.00		
	CG+GG	167(37.1)	218(50.7)	1.70 (1.25-2.27)	<0.001	
	Allele, G (%)	195(21.7)	272(31.7)			
rs10865710	Co- dominant					0.226
	CC	255(56.7)	234(54.4)	1.00		
	CG	161(35.8)	160(37.2)	1.07 (0.86-1.48)	0.628	
	GG	34(7.6)	36(8.4)	1.02 (0.70-1.69)	0.746	
	Dominant					
	CC	255(56.7)	234(54.4)	1.00		
	CG+GG	195(43.4)	196(45.6)	1.06 (0.87-1.51)	0.656	
	Allele, G (%)	229(25.4)	232(27.0)			
rs709158	Co- dominant					
	AA	263(58.4)	240(55.8)	1.00		0.836
	AG	161(35.8)	157(36.5)	1.02 (0.81-1.41)	0.434	
	GG	26(5.8)	33(7.7)	1.11 (0.78-1.62)	0.592	
	Dominant					
	AA	263(58.4)	240(55.8)	1.00		
	AA+GG	187(41.6)	190(44.2)	1.05 (0.80-1.45)	0.483	
	Allele, G (%)	213(23.7)	223(25.9)			

*Adjusted for gender, age, smoking and alcohol status, BMI, WC, FPG, TC, TG, HDL, educational year, prevalence of stroke, prevalence of diabetes.

GMDR analysis was used to investigate the impact of the interaction among 6 SNPs within *SORL1* and *PPAR G* gene on LOAD risk. Table 4 shows a significant two-locus model (p = 0.0010) involving rs1784933 and rs1805192, and in this model, the cross- validation consistency was 9/ 10, and the testing accuracy was 62.70%. We also found a significant two-locus model (p = 0.0100) involving rs1784933 and alcohol drinking, and in this model, the cross-validation consistency was 10/ 10, and the testing accuracy was 60.72%, after covariates adjustment for alcohol consumption status, FPG, TG and HDL (Table 5).

**Table 4:** Best gene–gene interaction models, as identified by GMDR

Locus no.	Best combination	Cross-validation consistency	Testing accuracy	p-values*
Gene- gene interaction				
2	rs1784933 rs1805192	9/10	0.6270	0.0010
3	rs1784933 rs1805192 rs10865710	8/10	0.5399	0.0547
4	rs1784933 rs1805192 rs10865710 rs3824966	7/10	0.5399	0.1719
5	rs1784933 rs1805192 rs10865710 rs3824966 rs689021	7/10	0.4958	0.3770
6	rs1784933 rs1805192 rs10865710 rs3824966 rs689021 rs709158	6/10	0.4958	0.4258
Gene- environment interaction				
2	rs1784933 alcohol drinking	10/10	0.6072	0.0100
3	rs1784933 rs1805192 alcohol drinking	8/10	0.5399	0.1719
4	rs1784933 rs1805192 rs10865710 alcohol drinking	7/10	0.5399	0.1719
5	rs1784933 rs1805192 rs10865710 rs3824966 alcohol drinking	6/10	0.4958	0.4258
6	rs1784933 rs1805192 rs10865710 rs3824966 rs689021 alcohol drinking	5/10	0.4958	0.3770
7	rs1784933 rs1805192 rs10865710 rs3824966 rs689021 rs709158 alcohol drinking	6/10	0.4958	0.9893

*Adjusted for gender, age, smoking and alcohol status, BMI, WC, FPG, TC, TG, HDL, educational year, prevalence of stroke, prevalence of diabetes.

**Table 5:** The D’ values among SNPs within *PPAR G* and *SORL1* gene for the linkage disequilibrium test

SNPs	D’ values	
*PPAR G* gene	rs10865710	rs709158
rs1805192	0.362	0.623
rs10865710	-	0.482
*SORL1* gene	rs3824966	rs689021
rs1784933	0.548	0.823
rs3824966	-	0.617

Pairwise LD analysis between SNPs was performed and D’ values were shown in Table 5, we found that just D′ value between rs1784933 and rs689021 within *SORL1* gene was 0.823, which shown a strong chain reaction. So we also conducted haplotype analysis between the two SNPs. We found that the most common haplotype in *SORL1* gene was rs1784933- G and rs689021- T haplotype, the frequency of which was 0.4701 and 0.5467 in case group and control group. Haplotype containing the rs1784933- A and rs689021- C alleles were associated with a statistically increased LOAD risk (OR = 1.86, 95%CI = 1.37– 2.52, *P* < 0.001) (Table 6).

**Table 6:** Haplotype analysis on association between *SORL1* gene and LOAD risk

Haplotypes	rs1784933	rs689021	Frequencies		OR (95%CI)	p-values*
			Case group	Control group		
H1	G	T	0.4701	0.5467	1.00	--
H2	A	T	0.2167	0.2131	1.17 (0.84– 1.68)	0.590
H3	G	C	0.2035	0.1921	1.28 (0.92 - 1.75)	0.612
H4	A	C	0.1097	0.0481	1.86 (1.37 – 2.52)	<0.001

*Adjusted for gender, age, smoking and alcohol status, BMI, WC, FPG, TC, TG, HDL, educational year, prevalence of stroke, prevalence of diabetes.

## DISCUSSION

In this study, we found that both the rs1784933- A allele and the rs1805192- G allele were associated with increased LOAD risk. However, we the others SNP within *SORL1* and *PPAR G* gene were not associated with LOAD susceptibility after covariates adjustment. Some studies have focused on the association between *PPAR G* and AD risk, however the results on this association were inconsistent. Some studies concluded different results in Finnish [19], Japanese [25] and Asians and Caucasians population [20], these studies indicated that SNP and haplotype analyses for *PPAR G* gene were not significant associated with AD risk, so they conclude that *PPAR G* did not related with AD in the Finnish population. However, some studies also confirmed a significant association of SNPs within *PPAR G* with AD [17, 18].

Although this was not the first association study focused on the SNP of *SORL1* polymorphism and the risk of LOAD risk, however, they also did not concluded consistent results. In 2007, Rogaeva et al [26] firstly reported an association between SNP in *SORL1* gene and AD incidence. From then on, several population- based studies were conducted for other populations. Minster et al [15] suggested no association with LOAD risk in their cohort. The data by Liu et al [27] also suggested the similar results on relationship between genetic variants in *SORL1* and the risk of AD. Some studies also found positive results on this association, which were similar with results obtained in current study. Kölsch et al [28] found that *SORL1* gene variants were associated with increased AD risk. Bettens et al [10] also indicated a significant association between common SNP within *SORL1* gene and LOAD, providing further evidence of genetic variations in *SORL1* affecting susceptibility of LOAD. In a Japanese population, Kimura et al [29] found that *SORL1* was genetically associated with Alzheimer disease, and the similar results were also obtained from the others studies [30- 32].

In this study, we found that LOAD risk was determined by both *SORL1* and PPAR G gene, and synergistic reaction of both gene and environmental factors, so we also conducted analysis on impact of gene- gene and gene- environment interaction on LOAD risk. We found a significant gene- gene interaction involving rs1784933 and rs1805192, and a significant gene- environment interaction involving rs1784933 and alcohol drinking. Previously, several environmental risk factors have been reported, including alcohol drinking [21, 22], which was associated with LOAD in this study. Previously just one study focused on the impact of interaction between *SORL1* rs2070045 polymorphism and *ApoE* genotype with the late-onset Alzheimer's disease, but they showed no interaction effect between *ApoE 4* and any of the rs2070045 genotypes. In this study we also conducted the haplotype analysis for the rs1784933 and rs689021 within *SORL1* gene, the D’ value of which was more than 0.8 (0.823), we found a haplotype containing the rs1784933- A and rs689021- C alleles were associated with a statistically increased LOAD risk.

The current study has some limitations, which should be considered. Firstly, limited number of SNPs in *SORL1* and *PPAR G* gene was included in this study, and in the future, more SNPs should be included in analysis. Secondly, more environmental factors should be included in the gene- environment analysis, not only for alcohol drinking. Thirdly, the results of interaction analysis should be checked in different population, not only in Chinese Han.

In conclusion, we found that rs1784933 and rs1805192 minor alleles were associated with increased LOAD risk. We also found a significant gene- gene interaction between rs1784933 and rs1805192, gene- environment interaction between rs1784933 and alcohol drinking, and haplotype containing the rs1784933- A and rs689021- C alleles were all associated with increased LOAD risk.
